# Inverted internal limiting membrane-flap technique for large macular hole: a microperimetric study

**DOI:** 10.1186/s40942-019-0195-6

**Published:** 2019-10-18

**Authors:** Giancarlo Sborgia, Alfredo Niro, Alessandra Sborgia, Valeria Albano, Tiziana Tritto, Luigi Sborgia, Valentina Pastore, Rossella Donghia, Ermete Giancipoli, Nicola Recchimurzo, Francesco Boscia, Giovanni Alessio

**Affiliations:** 1 Department of Medical Science, Neuroscience and Sense Organs, Eye Clinic, University of Bari, Bari, Italy; 2 Eye Clinic, Hospital “S. G. MOSCATI”, ASL TA, Via Per Martina Franca, 74010 Statte, Taranto Italy; 3 National Institute of Gastroenterology “S. de Bellis” Research Hospital, Castellana Grotte, Bari, Italy; 4 Department of Surgical, Microsurgical and Medical Sciences, Eye Clinic, University of Sassari, Sassari, Italy

**Keywords:** Large macular hole, Inverted ILM-flap, Microperimetry, Retinal sensitivity, Fixation behaviour

## Abstract

**Background:**

Inverted Internal Limiting Membrane (ILM)-flap technique would seem to lead to higher closure rate and better visual acuity than traditional procedure with ILM peeling for the treatment of large macular hole (LMH). Visual acuity recovery does not reveal many other functional changes related to surgical approach. Our purpose was to evaluate macular function and morphology over a 1-year follow-up after inverted ILM-flap technique for LMH by using microperimetry in order to predict visual prognosis.

**Methods:**

This study was a prospective unrandomized single-center study. 23 eyes of 22 patients with idiopathic LMH, with a minimum diameter ranging from 400 to 1000 μm, were included. All patients underwent vitrectomy with inverted ILM-flap technique and gas tamponade. We analyzed macular hole closure rate and functional outcomes including best-corrected visual acuity (BCVA), macular sensitivity (MS) at central 12° and central macular sensitivity (CMS) at central 4°, and fixation behavior as bivariate contour ellipse area (BCEA, degrees^2^) at 68%, 95%, and 99% of fixation points measured by microperimeter, over a follow-up of 12 months.

**Results:**

The macular hole closure rate was 98%. The BCVA improved from 20/230 (Logmar, 1.06 ± 0.34) to 20/59 (logMar, 0.47 ± 0.45) at last follow-up (p < 0.001). Retinal sensitivity and BCEA significantly improved (MS, p = 0.001; CMS, p < 0.0001; BCEA: 68%, p < 0.01; 95%, p < 0.01; 99%, p = 0.001). Multiple stepwise regression analysis showed the final BCVA was significantly associated with macular hole size (β = 0.002, p = 0.03), preoperative MS (β = − 0.06, p = 0.001) and BCEA at 95% and 99% of fixation points (β = − 0.12, p = 0.01; β = 0.06, p = 0.01).

**Conclusions:**

Inverted ILM-flap technique for LMH results in good morphologic and functional outcomes. Macular hole size and microperimetric parameters as preoperative MS and BCEA have a predictive role on post-surgical visual acuity.

## Background

Idiopathic full-thickness macular hole is an important cause of central vision impairment and metamorphopsia in elderly people [1]. The conventional procedure for macular hole surgery consists of internal limiting membrane (ILM) peeling and air or gas tamponade [2–5]. In cases with large macular hole (LMH) the anatomical success rate, as the closing of the hole, is as low as 40% to 80% using conventional procedure [6–8]. Michalewska et al., in a prospective trial, found that anatomical closure rate was higher using a novel technique of inverted ILM-flap technique (98%) compared to traditional procedure with ILM peeling (88%) for the treatment of LMH. In the same study post-operative visual acuity was significantly higher using the new technique [9]. Several papers have suggested that inverted ILM-flap technique may be better for the treatment of LMHs considering morphologic and functional outcomes [10–13]. Despite high closure rate and visual acuity recovery, functional changes after macular hole surgery are really complex. Visual acuity recovery does not reveal many other functional changes related to macular pathologies and surgical manipulations. So central retinal sensitivity and fixation behavior analysis using microperimeter can provide further objective and quantitative informations about macular function, enabling to analyse exact correlation between macular disease, as age-related maculopathy, diabetic macular oedema, macular oedema after vein occlusion, idiopathic epiretinal membrane, central serous chorioretinopathy, inflammatory macular oedema and macular dystrophy, and corresponding function, in assessment of natural history and treatment outcomes [14–22]. Previous papers reported a significant improvement of retinal sensitivity and fixation properties after traditional ILM peeling for macular hole [23–27], revealing a predictive role of preoperative macular hole feature and microperimetric parameters on visual recovery [27–30]. The aim of this study was to evaluate morphologic and functional outcomes after vitrectomy with inverted ILM-flap technique for LMH by using microperimetry in order to predict visual prognosis.

## Methods

In this prospective study we analysed 22 eyes of 23 consecutive patients with idiopathic LMH (diameter > 400 µ) who underwent 27-gauge vitrectomy with inverted ILM-flap technique. In all cases surgery was performed at the Eye Clinic of University of Bari, Bari, Italy, between April 2017 to April 2018. All the surgeries were performed by the same well-experienced retinal specialist (GS). Patients with amblyopia, severe refractive defect, corneal opacity, glaucoma or ocular hypertension, prior vitreoretinal surgery, proliferative diabetic retinopathy, retinal vascular disease, age-related macular degeneration, traumatic or myopic macular hole, retinal detachment due to macular hole, and minimum diameter of macular hole > 1500 μm were excluded. All patients underwent a complete ophthalmic examination, including best corrected visual acuity (BCVA) measurement (with ETDRS chart), slit lamp biomicroscopy, intraocular pressure (IOP) test, indirect ophthalmoscopy, optical coherence tomography (OCT) and microperimetry. BCVA was recorded as Snellen visual acuity and converted to logarithm of minimal angle of resolution (logMar) units for statistical analysis. Macular sensitivity and fixation stability were evaluated by MP-1 microperimeter (MP-1, Nidek Technologies, Padova, Italy). The MP-1 provides a 45° non-mydriatic view of the fundus with automated correction for eye movements. We performed microperimetry under room dim light condition. MP-1 uses a background luminance of 10 cd/m^2^, maximum stimulus intensity of 125 cd/m^2^, stimulus size of 0.11–1.73 degrees (Goldmann I–V), white stimulus colour, 0–20 dB dynamic range. Sensitivity was measured across a 45-point grid centered on the fovea using pattern Macula 12°–0 dB. At each point in the grid, sensitivity was measured for a white stimulus 0.438 in diameter (Goldmann size III) presented for 200 ms against a mesopic background (1.27 cd/m^2^). Threshold at each point was determined by using a 4-2 staircase. The ‘‘follow-up’’ feature of MP-1 was used to enable sensitivity measurements at the same retinal locations across all visits. Mean macular sensitivity (MS), the mean of all 45 loci in the central 12^°^ (1^°^ = 300 μm), and mean central macular sensitivity (CMS), the mean sensitivity of the central 13 loci (enclosed by a circle with a 4° diameter) were recorded. Fixation stability was recorded using MP-1 during the light sensitivity examination. The bivariate contour ellipse area (BCEA) values have been applied to obtain a quantitative measure of fixation stability [31]. We analysed BCEA which contains 68%, 95% and 99% of fixation points. We performed Spectral Domain OCT (RTVue™ Optovue, Inc, Fremont, CA, USA) using MM6 (6 mm × 6mm) scans and Cross Line HD (8 mm length) scans through the macula. Size of LMH was measured using the calliper function in the ‘retinal thickness analysis’ mode in the Optovue software. Macular hole closure rate was evaluated, defining hole closure as the flattening of macular hole with resolution of the subretinal cuff of fluid and neurosensory retina completely covering the fovea, confirmed by OCT scans. We investigated all patients before surgery and at month 1, 3, 6, 12 after surgery recording the values of those above parameters. After the purpose and procedures of the operation were explained, informed consent was obtained from all patients. All surgeries were performed under a retrobulbar block (a mixture of 2% Lidocaina and 2% Mepivacaina), using the Constellation vitrectomy system (Alcon, Fort Worth, TX, USA). Phacoemulsification was performed in all phakic eyes. All patients included in the study underwent 27-gauge transconjuctival sutureless vitrectomy with posterior hyaloid detachment. Then Brilliant blue G was used to stain ILM in the macula area. ILM peeling with inverted ILM-flap technique was adopted. The ILM peeling was done using pinch and grasp technique up to approximately 2 disc diameters around the macular hole. The edges of the ILM were trimmed with cutter and the remnant was then inverted to cover the macular hole. We lowered the perfusion pressure when inverting the flap and during air-fluid exchange. Gas tamponade with 22% SF6 was performed, and patients were instructed to remain in prone face positioning for 3 days postoperatively. The study followed the tenets of the Declaration of Helsinki and was approved by the institution’s review board.

Statistical analysis Statistical analysis was based on all patients included in the study. No formal sample size calculation was performed. Mean and standard deviations were used for continuous variables. A *t* test was performed on the changes from baseline in BCVA, MS, CMS and BCEA. All statistical tests were performed at the p < 0.05 significance level. Simple linear regression model was performed to assess the relationship between BCVA at 12 months and each indipendent variable. The independent variables included age, sex, lens status, axial length, baseline macular hole size, baseline BCVA, MS, CMS, and BCEA 68%, 95% and 99%. Multiple linear regression model in backward with stepwise method was performed to assess any predictive factors associated with postoperative visual acuity at 12 months (cut-off removal variable, p ≥ 0.10). The factors with a p value < 0.05 in the multiple model were considered as potential baseline predictors. Statistical analysis was made using STATA 12.1 Statistical Software (StataCorp), 2014, release 12 (College Station, TX).

## Results

**Fig. 1:**
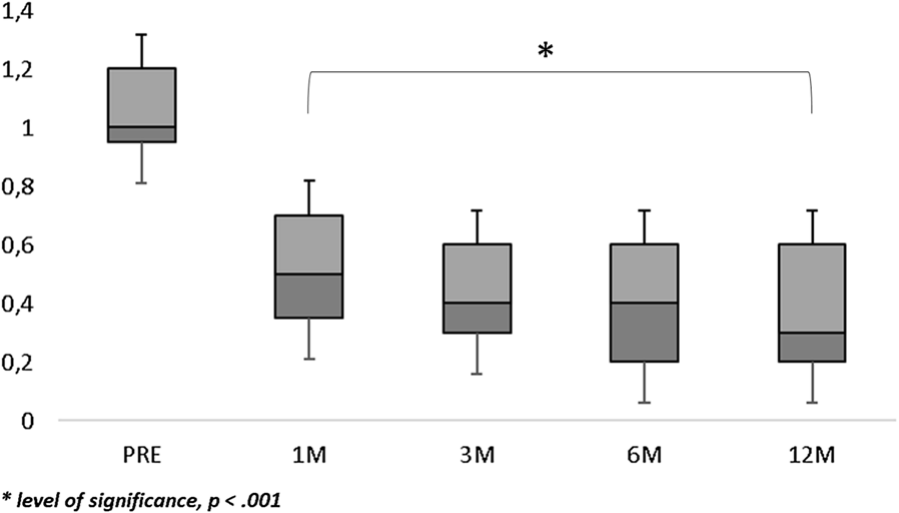
Visual acuity changes over follow-up. Visual acuity (Logmar) significantly improved after Internal Limiting Membrane (ILM)-flap inversion. Major visual acuity improvement was achieved at 1 and 3 months after surgery with a mild gain at 6 and 12 months

**Fig. 2:**
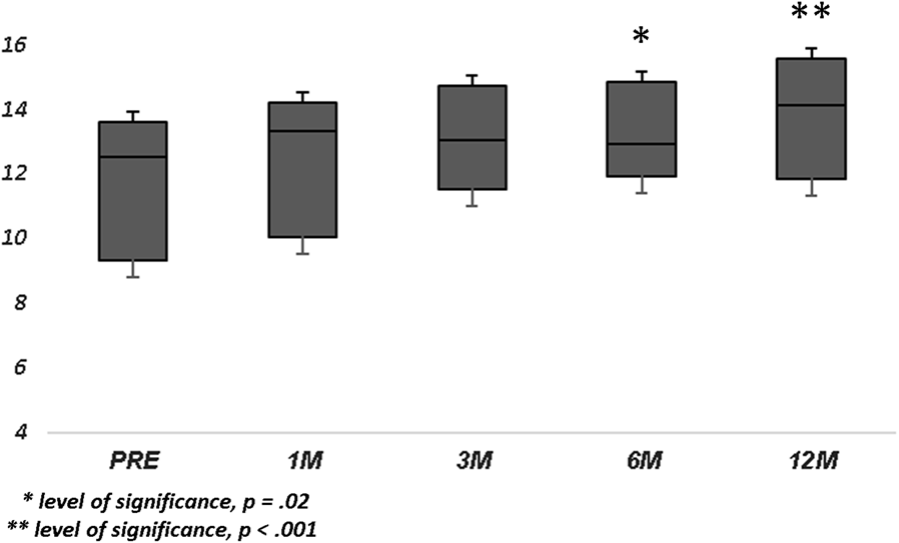
Retinal sensitivity changes over follow-up. Mean Macular Sensitivity (MS, decibels), meaning the mean of sensitivity of all 45 loci in the central 12°, significantly improved only at 6 and 12 months from surgery

**Fig. 3:**
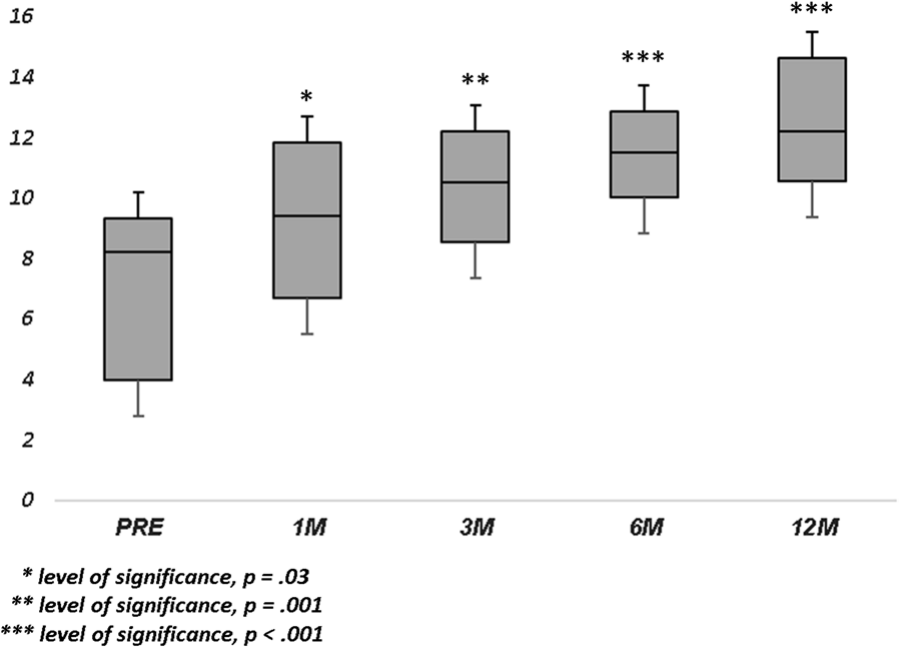
Central Macular Sensitivity changes over follow-up. Mean Central Macular Sensitivity (CMS, decibels), meaning the mean sensitivity of the central 13 loci (enclosed by a circle with a 4° diameter), improved incrementally from the first follow-up after surgery

**Fig. 4:**
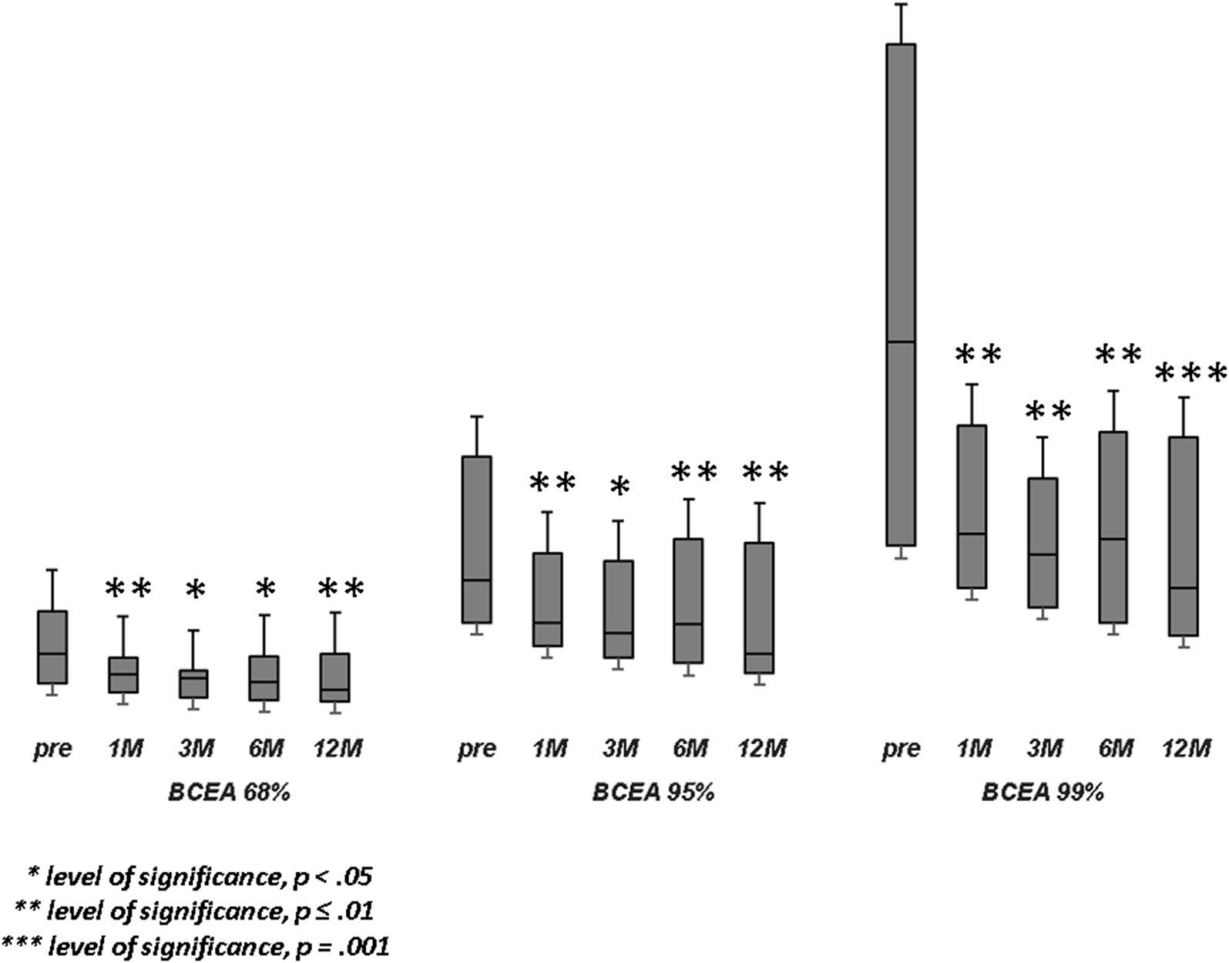
Fixation stability changes. The Bivariate Contour Ellipse Area (BCEA) values have been applied to obtain a quantitative measure of fixation stability. We analysed BCEA which contains 68%, 95% and 99% of fixation points. BCEA significantly decreased at all follow-up

**Fig. 5:**
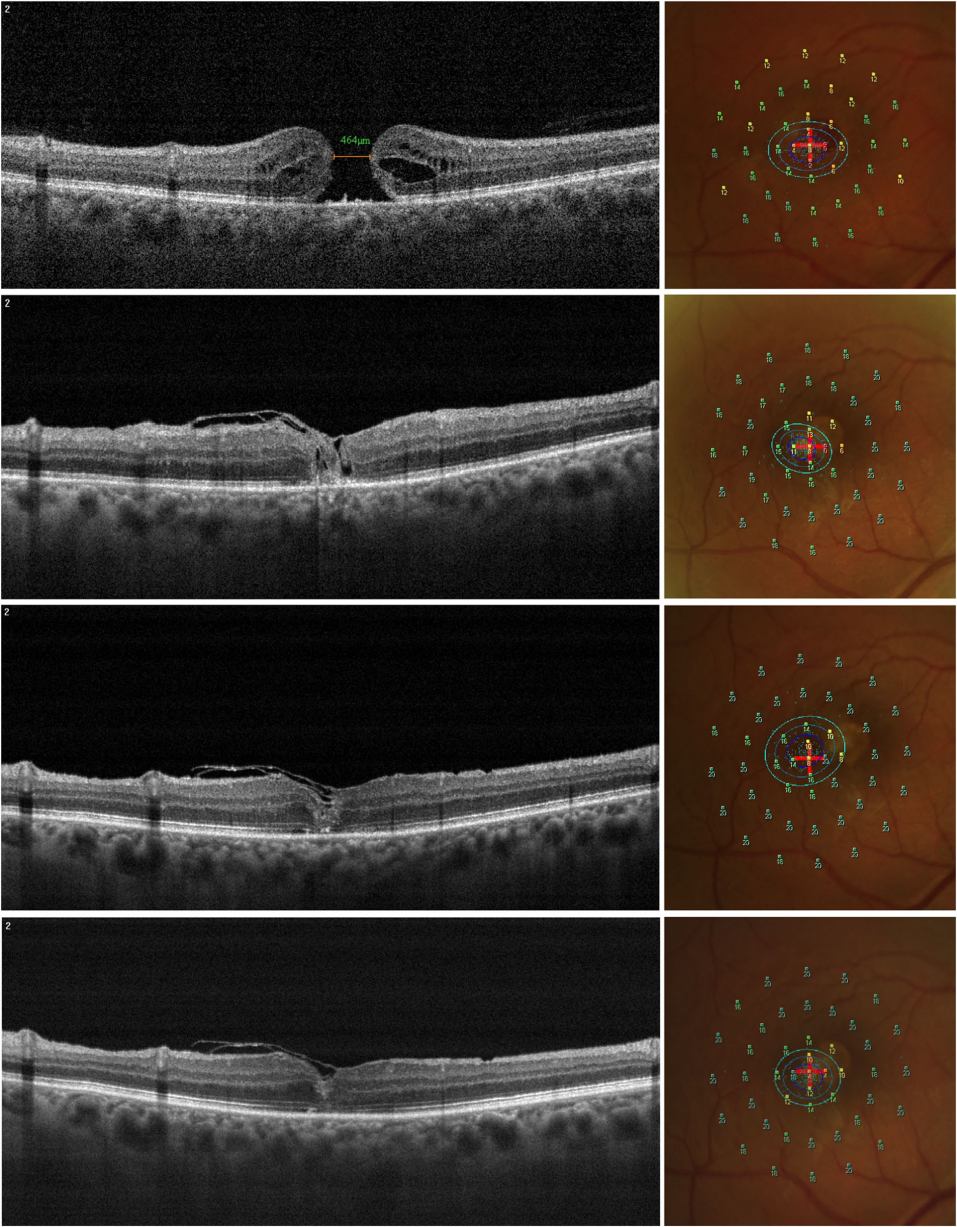
Representative case of inverted Internal Limiting Membrane (ILM)-flap technique for large macular hole (LMH). Morphologic and functional changes over follow-up. OCT scans show the resolution of macular hole over follow-up *(left column)*. Preoperative minimum macular hole diameter was 464 µm (*left column, top panel*). One month after surgery, the LMH was closed and the inverted ILM-flap could be seen covering the hole with a hyperreflective tissue inside the hole (*left column, second row panel*). Six months postoperatively, outer retinal layers were partially restored *(left column, third row panel).* Twelve months postoperatively, outer retinal layers were not completely restored *(left column, bottom panel).* Related microperimetric maps with Bivariate Contour Ellipse Area (BCEA) analysis over follow-up *(right column).* At baseline, microperimetry revealed an absolute scotoma (red/orange points with low sensitivity values) with a surrounding relative scotoma (yellow points) in the central degrees; BCEA (concentric ellipses) was large in diameter *(right column, top panel).* One month after surgery, both absolute and relative scotoma progressively reduced with a mild increase of whole sensitivity; BCEA showed a reduction in dimension *(right column, second row panel).* Six months postoperatively, all retinal sensitivity increased mainly on central degrees; BCEA had a mild increase in dimension *(right column, third row panel).* At last follow-up, we observed a mild reduction in central retinal sensitivity with a reduction of BCEA *(right column, bottom panel)*

Population’s characteristics are summarized in Table 1. A total of 23 eyes of 22 patients were included in this study. The mean age at surgery was 65.6 ± 5.7 years. Mean axial length was 23.32 ± 1.05 mm. Macular hole minimum diameter ranged from 402 µm to 1000 µm. All patients underwent 27-gauge vitrectomy with inverted ILM-flap technique and gas tamponade. The mean BCVA improved significantly from 20/230 (logMar, 1.06 ± 0.34) to 20/81 (logMar, 0.61 ± 0.41) at 1 month (p < 0.001), 20/62 (logMar, 0.49 ± 0.42) at 3 months (p < 0.001), 20/60 (logMar, 0.48 ± 0.43) at 6 months (p < 0.001), and 20/59 (logMar, 0.47 ± 0.45) at 12 months (p < 0.001) (Fig. 1). The mean MS improved from 11.30 ± 4.17 dB at baseline to 12.08 ± 4.11 dB at 1 month (p > 0.05), 12.35 ± 4.18 dB at 3 months (p > 0.05), 12.49 ± 4.23 dB at 6 months (p = 0.02), and 12.93 ± 4.38 dB at 12 months (p = 0.001) (Fig. 2). The mean CMS improved from 6.85 ± 3.87 dB at baseline to 8.70 ± 4.38 dB at 1 month (p = 0.03), 10.05 ± 4.86 dB at 3 months (p = 0.001), 11.09 ± 4.61 dB at 6 months (p < 0.001), and 11.74 ± 4.90 dB at 12 months (p < 0.001) (Fig. 3). BCEA changes at three different ellipses area which contain 68%, 95% and 99% of fixation points, significantly decreased at all follow-up (Table 2, Fig. 4). Simple linear regression analysis showed a significant relationship with each functional baseline parameter and last visual acuity. Multiple linear regression model on all variables together and a multiple linear regression model in backward with stepwise method revealed independent associations of baseline MS, BCEA at 95% and 99%, and macular hole size with final BCVA (Table 3). Mean IOP values were within normal range at baseline and at all post-surgery time points. Ocular hypertension (28 mmHg) was observed in only one patient 10 days after surgery. This condition was well controlled with local therapy (dorzolamide/timolol fixed combination 2 times/day). No other ocular or systemic complications were observed. A representative case is shown in Fig. 5.

**Table 1:** Baseline characteristics of patients (n = 22) and eyes (n = 23) underwent surgery

Age, years	
Mean (± SD)	65.6 ± 5.7
Range	51–72
Male: female	12:10
Axial length, mm	
Mean (± SD)	23.32 ± 1.05
Range	21.12–24.60
Lens status (phakic/pseudophakic)	9:14
MH size, µm	
Mean (± SD)	511 ± 129
Range	402–1000
IOP (mmHg)	14 ± 8
Baseline mean BCVA, Logmar	
Mean (± SD)	1.06 ± 0.34
Range	2.0–0.2
MS, dB	
Mean (± SD)	11.30 ± 4.17
Range	0.6–15.8
CMS, dB	
Mean (± SD)	6.85 ± 3.87
Range	0–12.8
BCEA, degree^2^ Mean (± SD)	
At 68%	5.10 ± 3.58
At 95%	12.66 ± 8.60
At 99%	22.68 ± 16.04

*SD* standard deviation, *MH* macular hole, *IOP* intraocular pressure, *BCVA* best corrected visual acuity, *logMAR* logarithm of minimum angle of resolution, *MS* macular sensitivity, *dB* decibel, *CMS* central macular sensitivity, *BCEA* biavariate contour ellipse area

**Table 2:** Changes of fixation quantitative parameter as Bivariate Contour Ellipse Area (BCEA, degree^2^) which contains 68%, 95% and 99% of fixation points

	Baseline	1 month	3 months	6 months	12 months
BCEA 68%, degree^2^	5.10 ± 3.58	3.37 ± 2.32	3.22 ± 2.74	3.40 ± 2.94	3.20 ± 2.97
BCEA 95%, degree^2^	12.66 ± 8.60	8.47 ± 5.36	8.28 ± 6.64	8.41 ± 6.04	7.68 ± 5.68
BCEA 99%, degree^2^	22.68 ± 16.04	14.83 ± 9.00	14.36 ± 11.58	14.73 ± 11.13	13.04 ± 9.76

**Table 3:** Linear regression model of best corrected visual acuity (BCVA) at 12 months on single variables (A); multiple linear regression model on all variables together in the model (B); final multiple linear regression model in backward with stepwise method (C)

Parameters^a^	β	se(β)	p-value	95% CI
(A)				
Sex (female/male)	− 0.32	0.18	0.09	− 0.70 to 0.05
Lens Status (phakic/pseuphakic)	0.35	0.18	0.07	− 0.03 to 0.72
Age	0.002	0.017	0.881	− 0.033 to 0.038
AL (mm)	0.06	0.09	0.52	− 0.13 to 0.25
MH size	0.001	0.001	0.087	− 0.0002 to 0.0027
BCVA pre (Logmar)	0.72	0.24	0.01	0.23 to 1.22
MS pre (dB)	− 0.067	0.017	0.001	− 0.102 to − 0.032
CMS pre (dB)	− 0.06	0.02	0.01	− 0.10 to − 0.01
BCEA 68% pre	0.081	0.021	0.001	0.037 to 0.124
BCEA 95% pre	0.03	0.01	0.01	0.01 to 0.05
BCEA 99% pre	0.014	0.005	0.016	0.003 to 0.025
(B)				
Sex (female/male)	0.07	0.16	0.67	− 0.28 to 0.42
LensStatus (Phakic/pseudophakic)	0.05	0.15	0.73	− 0.27 to 0.38
Age	0.004	0.011	0.69	− 0.019 to 0.003
AL (mm)	− 0.07	0.07	0.43	− 0.22 to 0.08
MH size	0.002	0.001	0.038	0.0001 to 000044
BCVA pre (Logmar)	0.26	0.27	0.35	0.335 to 0.862
MS pre (dB)	− 0.085	0.231	0.004	− 0.136 to 0.034
CMS pre (dB)	0.05	0.03	0.12	− 0.01 to 0.11
BCEA 68% pre	0.07	0.03	0.07	− 0.01 to 0.14
BCEA 95% pre	− 0.12	0.04	0.02	− 0.22 to 0.02
BCEA 99% pre	0.06	0.02	0.03	0.01 to 0.11
(C)				
MH size	0.002	0.001	0.029	0.0002 to 0.0036
MS pre (dB)	− 0.056	0.013	0.001	− 0.084 to − 0.028
BCEA 68% pre	0.05	0.03	0.10	0.01 to 0.11
BCEA 95% pre	− 0.11	0.04	0.01	− 0.20 to − 0.03
BCEA 99% pre	0.06	0.02	0.01	0.02 to 0.11

*β* coefficient, *se(β)* standard error of coefficient, *AL* axial length, *MH* macular hole, *BCVA pre* best corrected visual acuity preoperative, *Logmar* logarithm of minimum angle of resolution, *MS pre* retinal sensitivity preoperative, *dB* decibel, *CMS pre* central macular sensitivity preoperative, *BCEA 68%, 95%, 99%* bivariate contour ellipse area at 68%, 95%, and 99% of fixation points, respectively

^a^All variables included in the model were considered as continuous

## Discussion

The inverted ILM-Flap technique has been reported to have a high closure rate of LMH [7–11]. The ILM-flap might work as a scaffold for the proliferation and migration of activated Müller cells that promote the closure of macular hole producing neurotrophic factors [32]. This technique leads to an acceleration of the wound healing processes at the macula. We reported a high anatomical closure rate of 98%, in line with previous reports. In our experience, we found that some modifications of this technique, as lowering the perfusion pressure when inverting the flap and during air-fluid exchange, and the use of gas tamponade might help to avoid the dislocation of the ILM-flap during and after surgery increasing the closure rate, as confirmed by the results of previous papers [7, 8, 10–13]. The influence of flap inversion on functional recovery has been analysed in several papers to understand whether visual prognosis is affected by the presence of the flap over the hole. Indeed, the flap, working as a scaffold and basement membrane for tissue proliferation, should provide an environment to instruct the photoreceptors to assume correct position during the reconstruction process and finally to improve the postoperative visual acuity [9]. In line with previous studies, we found that postoperative visual acuity significantly improves after ILM-flap inversion [7–12]. In particular, major visual acuity improvement was achieved as early as one and 3 months after surgery, and no patients had clinically significant visual acuity improvement beyond this period. This trend in visual improvement could be achieved regardless the recovery of outer retinal layers at the foveal site that would seem occur after at least 3 months from surgery [33]. To date there are no definite conclusions on the role of inverted ILM-flap technique on the outer retinal layers changes and we did not analyse this point. However, our visual outcomes could be explained by the integrity of the detached photoreceptor layer in the perifoveal area or at the edges of the hole, and by the choice to cover rather than to fill the hole with the ILM-flap to avoid that glial tissue proliferation may mechanically obstruct the recovery of outer retinal layers, as previously suggested [34]. However, the functional effects of LMH and surgical technique could be underestimate by BCVA changes so microperimetry, a point to point measurement of retinal sensitivity, may better reflect the functional status and recovery after the hole has been repaired [23–26, 28, 35]. In this study, we recorded retinal sensitivity and fixation behavior as BCEA, before and after inverted ILM-flap technique for LMH over one-year follow-up. We analysed the changes in MS, which represents the general sensitivity within the central 12°, and CMS, indicating the retinal sensitivity within the central 4° of the macula. Previous studies suggested the necessity of investigating MS and CMS separately when evaluating central retinal sensitivity, considering that the sensitivity of the parafoveal retina area is higher than that at the central 0° in normal individuals, in part explained by the “masking effect” caused by the fixation target during examination [36, 37], and the age-related decrease of sensitivity in the perifoveal area than in the center of macula [38]. Moreover, our aim was to evaluate functional recovery at different retinal sites, at central 4° (corresponding to an area of 1200 µm in diameter at the foveal site) where the gliosis process, promoted by the inverted ILM-flap and controlled by Müller cells, could influence inner and outer retinal layers integrity with functional effects [9, 39, 40], and at central 12° where ILM peeling, inducing a temporary swelling of the arcuate nerve fiber layer (SANFL) as the earliest manifestation of dissociated nerve fiber layer (DONFL), leads to a reduced retinal sensitivity and paracentral scotomata in the peeled area [41]. At baseline, microperimetry demonstrated a lower sensitivity at central 4° (CMS) revealing an absolute scotoma, which corresponds to the neurosensory defect, with surrounding higher sensitivity at 12° (MS) defining a concentric relative scotoma in the region of the retina around the hole [42]. Our results showed that both CMS and MS significantly improved after surgery. CMS improved incrementally from the first follow-up, mainly at month 1 and 3 after surgery, probably related to the viability of detached photoreceptors at the edges of the hole [28, 29]. Instead, MS significantly improved only after 6 months from surgery, in particular nine patients showed an early reduction or unchanging of sensitivity at month 1 and 3, probably influenced by early functional damages on inner retinal layers due to ILM peeling, damages that are reversible as confirmed by our results and previous papers [41, 43]. Furthermore, Baba et al. observed that Brillant blue G-assisted vitrectomy could guarantee a faster restoration of IS/OS junction and a better postoperative BCVA and retinal sensitivity in the central degrees [44]. Fixation stability is another important functional parameter to consider in the treatment of macular diseases, probably more than fixation location in macular hole condition where the locus of fixation could already be naturally relocated out of the foveal site. We analysed the effect of inverted ILM-flap on a quantitative parameter of fixation behavior, as BCEA. In our series BCEA improvement had a similar trend at 68%, 95% and 99% of fixation points at all follow-up. We observed a reduction in dimension of the cloud of the fixation points at month 1 and 3, followed by a mild increase at 6 months and a new mild reduction of all ellipses after 12 months. Tarita-Nistor et al. reported the same improvement in fixation stability (BCEA) at 1 and 3 months after traditional ILM peeling [27]. As they suggested, the closure of the macular hole could lead to a complex reorganization of fixation behavior. Moreover, we can argue that changes in fixation stability would occur regardless the surgical technique used. In our experience, preoperative functional parameters evaluated as BCVA, MS, CMS and BCEA were individually correlated with final visual acuity. After that, a multiple stepwise linear regression analysis revealed that preoperative MS, BCEA at 95% and 99% of the fixation points and macular hole size have a predictive role on final visual acuity at 12 months. We suggest that MS has a significant influence on postsurgical visual acuity, probably because the inverted ILM-flap technique not always leads to photoreceptor reconstitution, and retinal sensitivity at 12° (MS) is less influenced by foveal microstructure recovery after macular hole closure than CMS at central 4°. The predictive role of a quantitative parameter of fixation behavior as BCEA on postsurgical visual acuity was already reported [27, 30], confirming that a smaller ellipsoid area correlates to more stable fixation and better visual performance. Also macular hole size was revealed as an independent factor predicting 12-month postoperative BCVA. Ota et al. observed the same predictive role of hole diameter for 6-month visual acuity in patients underwent different surgical techniques for LMH [45]. This could be because the recovery in foveal structure after inverted ILM-flap not always correspond to a complete reconstitution of outer retinal layers, not differently from traditional ILM peeling [45, 46]. Limitations of the study include the small sample size, the absence of a control group, the absence of analysis of outer retinal layers features at OCT scans related to functional changes, and the measurement error or intrinsic variability of microperimetric test. Factors acting on test variability are related to patient’s compliance and its anatomical and functional condition, and to the examiner and/or instrument. About patient condition, it should be mentioned the “learning factor” which can justify a certain degree of improvement during follow-up. Regards to the instrument, it should be mentioned the eye-tracker system, not able to ensure the same accuracy of analysis between the posterior pole and peripheral retina, the “point to point” overlapping error (0.5° to 2°) when “Follow-up” program is used, the “4-2 strategy” which can extend the duration of the test, the “ceeling effect” of MP-1, meaning the tendency to accumulate responses at the highest limit of the sensitivity threshold, and the size of the given stimulus (Goldmann III, 4 mm^2^ area, 26 min diameter of arc, or 0.4°) that, because of “spatial summation”, involves more photoreceptors which converge centrally on a single ganglional cell. As points of strength of this study we highlight the prospective nature of the study, the long term follow-up of 12 months and to our knowledge, the first microperimetric analysis of inverted ILM-flap technique involving different functional parameters.

## Conclusions

High closure rate and visual acuity improvement supported the effectiveness of the inverted ILM-flap technique for LMH. Macular sensitivity at central 12° and fixation analysis, detectable by microperimetry, give new informations on functional recovery, also revealing their predictive role on visual acuity after inverted ILM-flap technique.

## Data Availability

The datasets during and/or analysed during the current study available from the corresponding author on reasonable request.
